# Characterization and Classification of LMW-GS Genes at the *Glu-3* Locus of Bread Wheat

**DOI:** 10.3390/ijms262110482

**Published:** 2025-10-28

**Authors:** Yongying Zhao, Xianlin Zhao, Dan Zhang, Zhiguo Xiang, Hongshan Yang

**Affiliations:** Wheat Institute, Henan Academy of Agricultural Sciences (HAAS), Zhengzhou 450002, China

**Keywords:** bread wheat, LMW-GS genes, *Glu-3* loci, classification methods, sequence comparison

## Abstract

Low Molecular Weight Glutenin Subunits (LMW-GS) proteins have great effects on the end-use quality of bread wheat and are difficult to differentiate directly. It is very important to characterize and classify LMW-GS genes systematically. In this paper, 692 complete *Glu-3* gene sequences were retrieved from GenBank and were grouped based on their sequence characters and variations. Based on the characters of their N-terminal sequences, these genes were classified into two types, LMW-m and LMW-i, of which LMW-m genes were further classified into three sub-types based on their first amino acid (AA) (LMW-M, LMW-V and LMW-I). Based on the first seven or eight AA variations in the N-terminal sequence, LMW-GS *Glu-3* genes were classified into 16 types, namely LMW-N1 to LMW-N16. Based on the last 10 AA variations in the C-terminal, the *Glu-3* genes were classified into 22 types, designated as LMW-C1 to LMW-C22. Based on the number and distribution of cysteines, the *Glu-3* genes classified into 22 types included 7 conventional types with eight cysteines and 15 variant types with seven or nine cysteines. In addition, two new *Glu-A3* genes (*GluA-10* and *GluA-11*) were identified based on their sequence homology, and the connection between different classification methods was analyzed briefly. The results provide insight into the nature of the *Glu-3* gene family and are valuable for molecular marker-assisted selection of end-use quality traits in wheat improvement.

## 1. Introduction

Wheat is one of the most widely cultivated crops in the world, with more than 220 million ha planted annually under a wide range of climatic conditions and in many geographic regions [1]. Bread wheat (*Triticum aestivum* L., *n* = 7, AABBDD) flour has distinctive properties for forming doughs that are suited for the production of breads and lots of other important foods [2,3]. The processing properties of wheat flour are mainly affected by HMW-GS and LMW-GS proteins that form the disulphide-bonded gluten macropolymer [4,5,6] and contribute to the basic aspects of dough quality such as viscoelasticity and extensibility [7,8,9]. Therefore, HMW-GS and LMW-GS genes are essential for improving wheat’s end-use quality [10,11,12,13,14].

LMW-GS represents approximately one third of total seed storage proteins and 60% of the gluten fraction, and it has great effects on the end-use quality of bread wheat [15,16]. Most LMW-GS genes are encoded by the composite gene loci of *Glu-A3*, *Glu-B3* and *Glu-D3* on the short arms of the group 1 chromosomes of wheat [17,18,19,20], though LMW-GS loci on other chromosomes have also been reported, such as *Glu-B2* and *Glu-B4* on 1B [21,22], *Glu-D4* on 1D and *Glu-D5* on 7D [23]. Due to their clear resolution by gel electrophoresis and low gene copy number, the allelic variation in HMW-GS and its relation to wheat quality have been widely studied, and PCR-based DNA markers are available to differentiate the most important *Glu-1* alleles [24,25,26]. However, the resolution of gel electrophoresis-based LMW-GS analysis is low, and it is difficult to distinguish different alleles by analyzing the proteins directly because of their larger number of expressed subunits and their overlapping mobility with abundant gliadin proteins [27,28,29,30]. In view of this, the effects of individual LMW-GS genes on wheat quality are not very clear [31], and it is essential to characterize and categorize wheat LMW-GS genes and their deduced AA sequences.

A lot of LMW-GS genes or partial genes from bread wheat and its relatives have been cloned and registered in GenBank [32,33,34,35,36,37,38]. Zhao et al. cloned six different *Glu-D3* genes, identified 12 haplotypes in the six varieties and made a clear distinction among the subunits, coding genes, alleles and haplotypes of *Glu-D3* proteins [39,40]. Wang et al. identified six *Glu-B3* genes with 26 allelic variants and five *Glu-A3* genes with 19 allelic variations, respectively, and developed some DNA markers for their use in wheat breeding [41,42]. Based on the first AA presented in the N-terminal sequences of the glutenin subunits, three types of LMW-GS genes were identified, including LMW-s, starting with serine, LMW-I, starting with isoleucine, and LMW-m, starting with methionine [43,44,45,46]. Ikeda et al. further classified the LMW-GS genes into 12 groups according to their deduced amino acid sequences and, in particular, the number and position of cysteine residues [47], which could form intermolecular disulphide bonds [48]. Long et al. retrieved sixty-nine LMW-GS genes from GenBank and categorized them into nine groups using the deduced amino acid sequences of their highly conserved N-terminal domains [49]. With the increase in genes registered in GenBank, new types of *Glu-3* genes have been found, necessitating re-analysis of *Glu-3* gene sequences.

In our previous study, 146 *Glu-A3* genes, 136 *Glu-B3* genes and 311 *Glu-D3* genes with complete coding sequences were retrieved from GenBank, and they were classified into 9 *Glu-A3* genes with 69 allelic variants, 11 *Glu-B3* genes with 64 allelic variants and 10 *Glu-D3* genes with 96 variants, respectively, based on a homology comparison of nucleotide and deduced amino acid sequences [50]. In the present paper, more *Glu-3* genes were obtained from GenBank and were used to characterize and categorize LMW-GS genes systematically, as well as to verify and improve traditional classification methods. The results will be conducive to understanding LMW-GS genes more clearly and will provide a theoretical reference for the molecular marker-assisted improvement in wheat quality traits.

## 2. Results

The deduced amino acid sequences of 692 *Glu-3* genes with complete coding regions were compared, analyzed and classified based on the variations in their N-terminal sequences, C-terminal sequences and cysteine number and distribution. The results were as follows:

### 2.1. Classification of LMW-GS Genes Based on the Amino Acid Variations in Their N-Terminal Sequences

Based on the sequence variation in the N-terminal domain, two types of LMW-GS proteins were found among the 692 haplotypes, designated LMW-m and LMW-i, which accounted for 88.15% and 11.85% of the total, respectively (Table 1 and Table S1). Further analysis showed that the LMW-i type was relatively stable and had no variation at the first six AA loci in the N-terminal region. However, the LMW-m type had three allelic variants at the first AA locus, which were designed as LMW-M, LMW-I and LMW-V (using capital letters here to distinguish from LMW-m and LMW-i), accounting for 86.56%, 1.45% and 0.14%, respectively.

Based on the first seven or eight AAs in the N-terminal sequences, the LMW-m type was further divided into 15 sub-types, i.e., METSCIP, METSCIS, METSHIPG, METSHIPS, METSRVP, METRCIP, METRCVP, METSCIH, METSQIP, MDTSCIP, MDTSYIP, MEARCIP, MENSHIP, IENSHIP and VETSRVP, designed as LMW-N1 to LMW-N15 (shortened as N1 to N15 in this paper), respectively (Table 1 and Table S1). It was obvious that N6 was the most abundant variation type, accounting for 18.79%, followed by N5 (14.02%), N2 (13.01%), N13 (11.27%), N4 (10.12%) and N1 (8.38%). On the contrary, N7, N8, N12 and N15 were the least abundant LMW-m genes, each of which had only one variant in the 692 sequences. In addition, the fifth AA locus at the N-terminal was the most variable one with five allelic variations, compared with two or three mutations at the other AA loci (Table S1).

### 2.2. Classification of LMW-GS Genes Based on the Amino Acid Variations in C-Terminal Sequences

The sequence analysis of C-terminal peptides showed that there was no variation at the 11th AA (P) locus from the end, so the last 10 AAs were chosen for analysis of LMW-GS types. The grouping results presented that the 692 *Glu-3* genes were divided into 22 LMW-GS types, i.e., FGVGTGVGAY, FGVGAGVGAY, FGVGTGVGGY, FGVGTQVGAY, FGVGTRVGAY, FDVGTGVGAY, FGVDTGVGAY, FSVGTGVGAY, FGVSAGVGAY, FGVGSGVGAY, FGVGTGVSAY, FAVGTGVSAY, FGVGTGVGSY, FSIGTGVGAY, FSIGTGVGGY, LGVGSRVGAY, LGVGIGVGVY, LGVGIRVGVY, LGIGIGVGVY, LSIGTGVGGY, LGVGTGVGAY and LGVGIGVGXY, which were designated as LMW-C1 to LMW-C22 (here simplified as C1 to C22) (Table 2 and Table S2).

Among the 22 LMW-GS types, C1 was the predominant variation type, accounting for 33.82%, followed by C15 (11.56%), C5 (10.84%) and C17 (9.25%). Contrarily, C7, C9, C10, C12, C18, C20 and C21 were the least variable, with only one allelic variant in each group among the 692 sequences. Furthermore, the second AA from the last had the most variation (five), followed by the sixth and ninth AAs from the end, each having four variants. In addition, the last (Y) and the fourth (V) AAs backwards were very conserved, with no SNP found in the 692 *Glu-3* genes.

### 2.3. Classification of LMW-GS Genes Based on the Number and Distribution of Conserved Cysteine Residues

**Figure 1:**
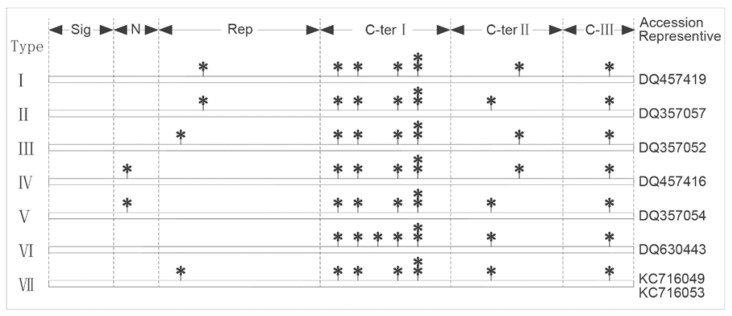
Classification of conventional LMW-GS sequences with 8 cysteines (*).

Based on the number and distribution of conserved cysteine residues, the 692 LMW-GS were classified into 22 types, including 7 conventional types with eight cysteines and 15 variation types with seven or nine cysteines (Figure 1 and Figure S1). Apart from the six conventional types (LMW-I to LMW-VI) named by Ikeda et al. [47], a new type (LMW-VII) was identified in this study. For the LMW-i type, all eight cysteines were located in the C-terminal domain and their locations were conserved, so only one type (VI) was identified. For the LMW-m type, seven cysteines were located in the C-terminal, and one was located in the N-terminal or in a repetitive region. Furthermore, the locations of the first cysteine in the repetitive region and the seventh cysteine in the C-terminal II were variable. Thus, a total of six types were found. Among them, LMW-IV and LMW-V were the most abundant types, and they accounted for 25.29% and 20.09%, respectively. And LMW-VII was the least variable type, with only two haplotypes identified in the 692 sequences (Table 3).

**Figure 2:**
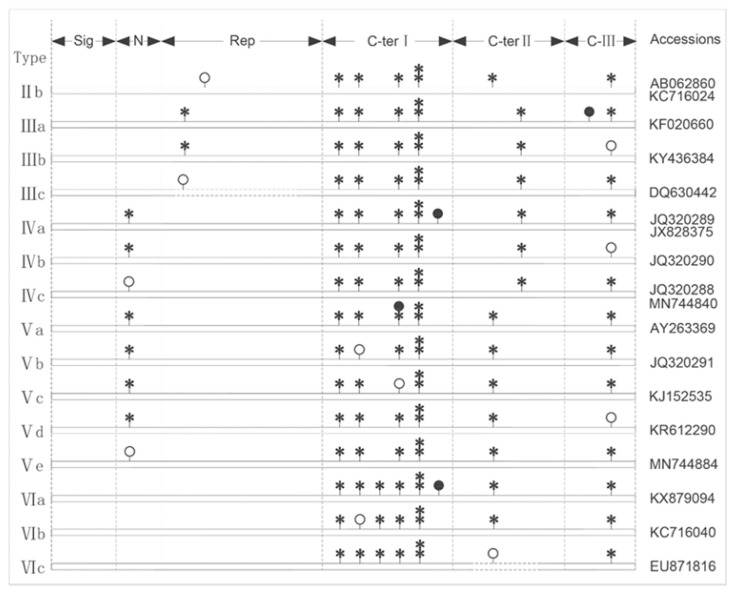
Classification of variation in LMW-GS sequences with 7 or 9 cysteines. Asterisks present the locus of original cysteines; a black circle presents the locus of an extra cysteine; and a white circle presents the locus of a missing cysteine.

Among the 15 variation types with seven or nine cysteines, 13 were caused by cysteine mutations (SNP) and 2 by sequence fragment (Indel; IIIc and VIc). Therefore, they were named as LMW-IIb, IIIa, IIIb, IIIc, IVa, IVb, IVc, Va, Vb, Vc, Vd, Ve, VIa, VIb and VIc on the basis of conventional types, of which 4 genes had nine cysteines (IIIa, IVa, Va and VIa) and the other 11 had seven cysteines (Figure 2 and Figure S1). LMW-II, III, IV, V and VI had one, three, three, five and three variant types, respectively, and no variation was found in LMW-I and LMW-VII. The 15 variant types included only 18 haplotypes, accounting for 2.6% of the 692 sequences.

### 2.4. Association Analysis of Glu-3 Genes Among Different Classification Methods

In our previous study, 9 *Glu-A3*, 10 *Glu-B3* and 11 *Glu-D3* genes with complete coding regions were named, respectively, by means of a homology comparison of nucleotide and AA sequences [50]. In this study, two new *Glu-A3* genes were added, designated as *GluA3-10* (JX878001, JX878048 and JX878064) and *GluA3-11* (JX877796 and JX877797), which presented an AA similarity of less than 91% and 98% with the other 9 *Glu-A3* genes, respectively (Table 4, Tables S1 and S2). The 489 located *Glu-3* genes (excluding pseudogenes) were selected to conduct an association analysis of N-terminal and C-terminal sequences. It was found that each *Glu-3* gene contained 1-3 C-terminal types and 1-3 N-terminal types except *GluD3-2*, which had 5 N-terminal types. In addition, the *Glu-B3* genes had relatively fewer variant types, including four N-terminal types (N4, N5, N9 and N13) and six C-terminal types (C1, C3, C5, C13, C14 and C15). But the *Glu-D3* genes had the most variant types, including 11 N-terminal types and 10 C-terminal types (Table 4). Based on the classification of cysteine location, *Glu-A3*, *Glu-B3* and *Glu-D3* genes had four, three and five types of LMW-GS proteins, respectively (Table 3). This indicates that the *Glu-D3* genes coded the most diverse LMW-GS proteins.

Further analysis showed that N1 had the most C-terminal variations (eight), followed by N6 (six), N16 (six), N2 (five) and N13 (five). This suggests that these were the major LMW-GS types. Similarly, C1 represented the most N-terminal variations (10), followed by C5 (4) and C6 (4), indicating that they were also among the major LMW-GS types (Table 4). Interestingly, all 66 LMW-N16 types presented at the *Glu-A3* locus and belonged to the LMW-i type (Table 3 and Table S3).

## 3. Discussion

The wheat LMW-GS represents a special protein family that is mainly coded by more than 30 genes at *Glu-A3*, *Glu-B3* and *Glu-D3* loci [50]. In this study, 692 complete *Glu-3* gene sequences were retrieved from GenBank and were grouped based on their sequence characters and variations with the purpose of gathering insight into the nature of the *Glu-3* gene family. This is helpful for understanding the end-use quality traits in wheat improvement.

### 3.1. Characteristics of LMW-GS Genes and Their Structural Domain Variations

The differentiation of proteins and scoring of alleles by direct analysis of LMW-GS genes was extremely difficult because of their high similarity, repeatability and complexity [40,42]. It is very important to characterize and categorize the LMW-GS genes and their deduced AA sequences to understand their functions and improve wheat end-use quality.

The deduced AA sequences indicated that all the 692 LMW-GS had five typical domains, i.e., N-terminal conservation, repetition, C-terminal I (cysteine-rich), C-terminal II (glutamine-rich) and C-terminal III (conserved domain) [40]. Among them, 674 genic sequences showed a typical eight conserved cysteine residues, and 18 genic sequences had seven or nine cysteine residues [51,52,53,54,55], which were from AA mutation or fragment deletion. Based on the number and distribution of cysteines [47,56], they were classified into 22 types, including 7 conventional types and 15 variant types. It was interesting that the locations of the eight cysteines were well regulated. For the LMW-i type, all eight cysteines were found in the C-terminal region and their locations were conserved, so there was only one type of LMW-i (LMW-VI). For LMW-m, six cysteines had fixed locations, but the first cysteine presented three location variations, one located in the N-terminal conservation region and two in the repetitive region. The seventh cysteine in the C-terminal region also had two locus variations. Therefore, the LMW-m peptides had six types in all (Figure 1).

It is generally recognized that the first and seventh cysteine seem to be involved in the formation of intermolecular disulphide bonds [57], and the additional cysteine may offer more opportunity for the LMW-GS proteins to form larger polymeric proteins, which would contribute to dough elasticity [51]. The studies of Dong et al. show that the ratio of α-helix plus β-folding in the secondary structure of LMW-GS genes with seven cysteines was obviously higher than those with eight cysteines [58]. This suggests that the 15 variant types may be beneficial to change the tertiary structure of peptides, and then may affect the protein function and flour quality of wheat.

### 3.2. Classifications of LMW-GS Genes and Their Association Analysis

Although several classification methods have been proposed in previous studies [28,46,56], their classification results may be outdated now because of the limited number of analyzed sequences. It was widely accepted that there were three types of LMW-GS genes based on the first AA of deduced peptides, LMW-m, LMW-s and LMW-i [32,43,44,45]. However, the LMW-s type was not found among the 692 *Glu-3* genic sequences. The sequence analysis indicated that LMW-s should be an incomplete gene (i.e., partial gene), which may be from the deletion of the first three AAs of the N-terminal (MET or MDT; Table 1 and Table S1).

In fact, the two types of LMW-GS (LMW-m and LMW-i) should be named based on the sequence characters of the N-terminal domain, rather than based on the first AA. When based on the first AA, LMW-m could be further divided into three sub-types, LMW-M, LMW-V and LMW-I (the latter two should be mutation types of LMW-M), and LMW-i had no variant type. In addition, LMW-m could be further classified into 15 types based on the first seven or eight AAs of the N-terminal, much more than the reported types by Ikeda et al. [47]. This indicates that the AA variation in the N-terminal region was more diverse than imagined.

As for the classification based on the AA variations in the C-terminal sequences, no systematic research result was reported before, though some works were conducted by Ikeda et al. [56]. In this paper, a total of 22 types were found based on the last 10 AAs of the C-terminal region (LMW-C1 to LMW-C22). Among them, LMW-C1 was the main type, accounting for 33.82%, and 2–5 allelic variations were found at each AA locus except the first, fourth and eleventh locus from the end, which were conservative and had no AA variation in any of the 692 deduced peptides. This indicates that the conservativeness in the C-terminal domain was also relative.

It was found that these *Glu-3* genes showed a certain connection among different classification methods. For instance, all 66 LMW-N16 types presented at the *Glu-A3* locus and belonged to LMW-i type (Table 3, Figure S1), and all *GluD3-5*, *GluD3-3* and *GluD3-1* genes belonged to the LMW-I, -II and -III types, respectively. Each of the *Glu-3* genes contained 1-3 C-terminal types and 1-3 N-terminal types except *GluD3-2*, which had 5 N-terminal types. In addition, no LMW-I, LMW-II or LMW-III types were found in the *Glu-A3* genes, no LMW-IV, LMW-V and LMW-VI types were found in the *Glu-B3* genes, and the locus of the LMW-VII type was unclear. It was obvious that the *Glu-D3* genes had the most variant types, including 11 N-terminal types and 10 C-terminal types (Table 4). The variant types of LMW-GS indicate the complex regulatory mechanisms of wheat adaptability under the pressures combined by different agroecological zones and end-use quality. These results are beneficial to understanding the complex factors determining wheat quality.

### 3.3. Application Prospects of LWM-GS in Wheat Quality Breeding

LMW-GS genes play a pivotal role in determining wheat end-use quality [51]. Characterizing LMW-GS genes and their deduced amino acid (AA) sequences is therefore essential for deciphering gluten formation mechanisms and guiding wheat quality improvement breeding. However, direct analysis of LMW-GS for protein differentiation and allele scoring remains highly challenging due to the high similarity, repetitiveness and complexity of the *Glu-3* genes [40,41,42], which have received considerable attention from wheat researchers.

This study presents the importance of characterizing the LMW-GS genes and their allelic variations, and the results indicate that the LMW-GS genes were more complicated than expected. Though earlier studies have indicated that some alleles of LMW-GS are clearly more beneficial or detrimental on dough properties than others [6,7,8,20], and different molecular markers have been developed base on some identified *Glu-3* gene sequences [11,40,41], the function of individual LMW-GS genes in the determination of wheat quality is still less clear.

The results of this study should be helpful for the systemic identification and characterization of more LMW-GS genes for the development of functional markers to improve the selection efficiency of wheat quality breeding.

## 4. Materials and Methods

### 4.1. Sequence Collection and Alignment of LMW-GS Genes

The LMW-GS gene sequences of wheat located at the three *Glu-3* loci were retrieved from the NCBI (National Center for Biotechnology Information, www.ncbi.nlm.nih.gov; accessed before 10 January 2025). A total of 692 *Glu-3* gene sequences with complete coding regions were obtained, including 489 located and 203 unlocated. Pseudogenes were excluded from subsequent analyses. All retrieved DNA and protein sequences were aligned and analyzed using DNAman software, version 9.0 (Lynnon Biosoft, San Ramon, CA, USA) with default parameters.

### 4.2. Classification of LMW-GS Gene

The deduced amino acid sequences of all 692 LMW-GS genes were compared, and classification was performed according to sequence variation patterns among various *Glu-3* loci. The serial numbers of the *Glu-A3*, *Glu-B3* and *Glu-D3* genes were assigned a serial number based on the sequence homology established by Zhao et al. (2025) [50]. To further characterize the structural diversity of the LMW-GS proteins, the N-terminal and C-terminal regions were classified based on the sequence variations in the N-terminal domain and C-terminal sequences according to the methods proposed by Kasarda et al. [43,44] and Ikeda et al. [47], respectively. Additionally, the number and positional distribution of cysteine residues were analyzed and used as classification indicators following the methods proposed by Ikeda et al. [47] and Long et al. [49].

## 5. Conclusions

In this study, 692 *Glu-3* genic sequences with complete coding domains were retrieved from GenBank, and their sequence characters and variations were compared and analyzed systematically. On this basis, the traditional classification methods and results of LMW-GS genes were revised and supplemented. In addition, the connection between different classification methods was discussed. The results may be useful for understanding the diverse nature of LMW-GS genes at *Glu-3* loci and will be of help in systemically identifying and characterizing more LMW-GS genes for the development of functional markers to improve the selection efficiency for quality wheat breeding.

## Figures and Tables

**Table 1:** The first 7 or 8 AA variations in N-terminal sequences.

LMW Types of N-Terminal Domain			1	2	3	4	5	6	7	8	Gene Numbers			
											Located	Unknown	Total	%
LMW-m	M	N1	M	E	T	S	C	I	P	-	46	12	58	8.38
		N2	M	E	T	S	C	I	S	-	55	35	90	13.01
		N3	M	E	T	S	H	I	P	G	34	4	38	5.49
		N4	M	E	T	S	H	I	P	S	56	14	70	10.12
		N5	M	E	T	S	R	V	P	-	56	41	97	14.02
		N6	M	E	T	R	C	I	P	-	79	51	130	18.79
		N7	M	E	T	R	C	V	P	-	1	0	1	0.14
		N8	M	E	T	S	C	I	H	-	1	0	1	0.14
		N9	M	E	T	S	Q	I	P	-	3	0	3	0.43
		N10	M	D	T	S	C	I	P	-	22	8	30	4.33
		N11	M	D	T	S	Y	I	P	-	1	1	2	0.29
		N12	M	E	A	R	C	I	P	-	1	0	1	0.14
		N13	M	E	N	S	H	I	P	-	59	19	78	11.27
	I	N14	I	E	N	S	H	I	P	-	8	2	10	1.45
	V	N15	V	E	T	S	R	V	P	-	1	0	1	0.14
LMW-i		N16	I	S	Q	Q	Q	Q	-	-	66	16	82	11.85
Summation		16	3	2	3	2	5	2	2	2	489	203	692	100

The numbers in the last row of the columns from 4th to 11th did not include the LMW-i type.

**Table 2:** The last 10 AA variations in C-terminal sequences.

Types of C-ter.	11	10	9	8	7	6	5	4	3	2	1	Gene Numbers			
												Located	Unknown	T	%
LMW-C1	P	F	G	V	G	T	G	V	G	A	Y	159	75	234	33.82
LMW-C2	P	F	G	V	G	A	G	V	G	A	Y	30	16	46	6.65
LMW-C3	P	F	G	V	G	T	G	V	G	G	Y	15	8	23	3.32
LMW-C4	P	F	G	V	G	T	Q	V	G	A	Y	32	4	36	5.20
LMW-C5	P	F	G	V	G	T	R	V	G	A	Y	60	15	75	10.84
LMW-C6	P	F	D	V	G	T	G	V	G	A	Y	18	9	27	3.90
LMW-C7	P	F	G	V	D	T	G	V	G	A	Y	0	1	1	0.14
LMW-C8	P	F	S	V	G	T	G	V	G	A	Y	0	5	5	0.72
LMW-C9	P	F	G	V	S	A	G	V	G	A	Y	0	1	1	0.14
LMW-C10	P	F	G	V	G	S	G	V	G	A	Y	3	1	4	0.58
LMW-C11	P	F	G	V	G	T	G	V	S	A	Y	0	3	3	0.43
LMW-C12	P	F	A	V	G	T	G	V	S	A	Y	0	1	1	0.14
LMW-C13	P	F	G	V	G	T	G	V	G	S	Y	8	5	13	1.88
LMW-C14	P	F	S	I	G	T	G	V	G	A	Y	8	9	17	2.46
LMW-C15	P	F	S	I	G	T	G	V	G	G	Y	49	31	80	11.56
LMW-C16	P	L	G	V	G	S	R	V	G	A	Y	43	3	46	6.65
LMW-C17	P	L	G	V	G	I	G	V	G	V	Y	51	13	64	9.25
LMW-C18	P	L	G	V	G	I	R	V	G	V	Y	1	0	1	0.14
LMW-C19	P	L	G	I	G	I	G	V	G	V	Y	10	1	11	1.59
LMW-C20	P	L	S	I	G	T	G	V	G	G	Y	0	1	1	0.14
LMW-C21	P	L	G	V	G	T	G	V	G	A	Y	0	1	1	0.14
LMW-C22	P	L	G	V	G	I	G	V	G	X	Y	2	0	2	0.29

**Table 3:** The statistical table of seven types of LMW-GS genes based on the cysteine number and loci.

	Types	I	II	III	IV	V	VI	VII	Total
Location									
*GluA3*-	S/N	-	-	7	2, 5	6	1, 4, 10, 11	-	101
	Num.	0	0	2	20 + 1	12	64 + 2	0	
*GluB3*-	S/N	4, 11	1, 2, 3, 5, 6	8, 9	-	-	-	-	101
	Num.	59	34	6 + 2	0	0	0	0	
*GluD3*-	S/N	5	3	1	4, 6, 8	2, 7	-	-	287
	Num.	34	33	46 + 1	98 + 2	69 + 4	0	0	
Unlocated	Num.	18	18 + 2	39	52 + 2	53 + 1	15 + 1	2	203
Total	Num.	111	87	96	175	139	82	2	692
%		16.04	12.57	13.87	25.29	20.09	11.85	0.29	100

The number before and after the plus sign means “conventional types” + “variation types”.

**Table 4:** Association analysis of 489 located *Glu-3* genes between N-terminal types and C-terminal types.

Genes		Types of C-Terminal		Types of N-Terminal		Representation of Accessions	Total
		AA Sequence	No.	AA Sequence	No.		
*GluA3*-	1	C17: LGVGIGVGVY	48	N16: ISQQQQ	49	AY453154; FJ549928, et al.	101
		C18: LGVGIRVGVY	1			KR612276.	
	2	C1: FGVGTGVGAY	20	N10: MDTSCIP	18	AB062868; FJ549937; et al.	
				N11: MDTSYIP	1	JQ320288.	
				N2: METSCIS	1	JQ320292.	
	4	C17: LGVGIGVGVY	2	N16: ISQQQQ	2	X877798; JX877977.	
		C19: LGIGIGVGVY	10		10	AB062877; FJ549945; et al.	
	5	C1: FGVGTGVGAY	1	N10: MDTSCIP	1	FJ549946.	
	6		11	N6: METRCIP	11	MG574321; MN744845; et al.	
			1	N12: MEARCIP	1	MG574341.	
	7	C15: FSIGTGVGGY	2	N5: METSRVP	2	DQ357052; MG574328.	
	10	C1: FGVGTGVGAY	3	N16: ISQQQQ	3	JX878001; JX878048; et al.	
	11	C22: LGVGIGVGXY	2		2	JX877796; JX877797.	
*GluB3*-	1	C1: FGVGTGVGAY	6	N13: MENSHIP	6	EU369699; MH347497; et al.	101
	2	C13: FGVGTGVGSY	8		8	EU369704; JX163861; et al.	
	3	C3: FGVGTGVGGY	7		7	EU369715; FJ755309; et al.	
	4	C5: FGVGTRVGAY	55	N4: METSHIPS	56	AB062852; EU369719; et al.	
		C1: FGVGTGVGAY	1			FJ972196.	
	5		7	N13: MENSHIP	7	AB262661; EU369706; et al.	
	6	C3: FGVGTGVGGY	6		6	EU369711; JX877832; et al.	
	8	C14: FSIGTGVGAY	7	N5: METSRVP	7	DQ630441; KF020661; et al.	
	9		1		1	KF020660.	
	11	C5: FGVGTRVGAY	3	N9: MET-SQIP	3	JX877826; JX8779942; et al.	
*GluD3*-	1	C15: FSIGTGVGGY	47	N5: METSRVP	46	DQ357052; FJ755313; et al.	287
				N15: VETSRVP	1	KR612285.	
	2	C1: FGVGTGVGAY	55	N1: METSCIP	1	JQ796685.	
				N10: MDTSCIP	1	JQ796690.	
				N8: METSCIH	1	JQ796686.	
				N6: METRCIP	52	DQ357054; JX877783; et al.	
		C17: LGVGIGVGVY	1		1	KR612291.	
		C6: FDVGTGVGAY	16		15	AY263369; DQ357056; et al.	
				N7: METRCVP	1	FJ172533.	
	3	C3: FGVGTGVGGY	2	N13: MENSHIP	2	KR612293; KR612294.	
		C2: FGVGAGVGAY	30		23	DQ357057; JF339167; et al.	
				N14: IENSHIP	7	DQ357058; FJ755316; et al.	
		C6: FDVGTGVGAY	1		1	EU189095.	
	4	C1: FGVGTGVGAY	53	N2: METSCIS	54	DQ457416; EU189093; et al.	
		C6: FDVGTGVGAY	1			HM055909.	
	5	C4: FGVGTQVGAY	32	N3: METSHIPG	34	AB062851; DQ457419; et al.	
		C5: FGVGTRVGAY	2			FJ755317; KR612298.	
	6	C16: LGVGSRVGAY	43	N1: METSCIP	44	DQ457420; EU189091; et al.	
		C10: FGVGSGVGAY	1			EU189090.	
	7	C1: FGVGTGVGAY	1		1	EU189092.	
	8	C10: FGVGSGVGAY	2	N10: MDTSCIP	2	JQ320289; JQ796687; et al.	
Total		-	489	-	489	-	489

The pseudogenes were not included. *GluA3-10* and *GluA3-11* were newly named in this paper.

## Data Availability

The LMW-GS gene sequences of wheat at three *Glu-3* loci were searched at NCBI (National Centre for Biotechnology Information, www.ncbi.nlm.nih.gov. Accessed on 9 January 2025).

## Supplementary Materials

The following supporting information can be downloaded at https://www.mdpi.com/article/10.3390/ijms262110482/s1.

## Abbreviations

The following abbreviations are used in this manuscript:

LMW-GS	Low-molecular-weight glutenin subunit
AA	Amino acid
Glu	Glutenin
