# Supplementary material for: Characterization and Classification of LMW-GS Genes at the Glu-3 Locus of Bread Wheat
Source: Int J Mol Sci. 2025 Oct 28;26(21):10482. doi: 10.3390/ijms262110482 (PMC12610552; doi:10.3390/ijms262110482)
Supplement: Supplementary file 1 [file ijms-26-10482-s001.zip › Table S3.pdf]

Table S3. The correspondence table of N-terminal types and C-terminal types for LMW-GS genes.

Types		N1	N2	N3	N4	N5	N6	N7	N8	N9	N10	N11	N12	N13	N14	N15	N16	Total	
		MET SCIP	MET SCIS	MET SHIPG	MET SHIPS	MET SRVP	MET RCIP	MET RCVP	MET SCIH	MET SQIP	MDT SCIP	MDT SYIP	MEA RCIP	MEN SHIP	IEN SHIP	VET SRVP	ISQ QQ-	Types	No.
C1	FGVGT GVGAY	2/3	53/29	-	1/0	-	64/31	-	1/0	-	20/7	1/1	1/0	13/3	-	-	3/1	10	159/75
C2	FGVGA GVGAY	-	-	-	-	-	0/7	-	-	-	-	-	-	23/7	7/2	-	-	3	30/16
C3	FGVGT GVGGY	-	-	-	-	-	0/2	-	-	-	-	-	-	15/6	-	-	-	2	15/8
C4	FGVGT QVGAY	0/1	-	32/3	-	-	-	-	-	-	-	-	-	-	-	-	-	2	32/4
C5	FGVGT RVGAY	-	0/1	2/0	55/14	-	-	-	-	3/0	-	-	-	-	-	-	-	4	60/15
C6	FDVGT GVGAY	-	1/1	-	-	-	15/8	1/0	-	-	-	-	-	-	1/0	-	-	4	18/9
C7	FGVDT GVGAY	-	-	-	-	-	-	-	-	-	0/1	-	-	-	-	-	-	1	0/1
C8	FSVGT GVGAY	0/1	0/1	-	-	-	0/3	-	-	-	-	-	-	-	-	-	-	3	0/5
C9	FGVSA GVGAY	-	-	-	-	-	-	-	-	-	-	-	-	0/1	-	-	-	1	0/1
C10	FGVGS GVGAY	1/1	-	-	-	-	-	-	-	-	2/0	-	-	-	-	-	-	2	3/1
C11	FGVGT GVSAY	-	0/3	-	-	-	-	-	-	-	-	-	-	-	-	-	-	1	0/3
C12	FAVGT GVSAY	0/1	-	-	-	-	-	-	-	-	-	-	-	-	-	-	-	1	0/1
C13	FGVGT GVGSY	-	-	0/1	-	0/2	-	-	-	-	-	-	-	8/2	-	-	-	3	8/5
C14	FSIGT GVGAY	0/1	-	-	-	8/8	-	-	-	-	-	-	-	-	-	-	-	2	8/9
C15	FSIGT GVGGY	-	-	-	-	48/30	-	-	-	-	-	-	-	-	-	1/0	0/1	3	49/31

C16	LGVGSRVGAY	43/3	-	-	-	-	-	-	-	-	-	-	-	-	-	-	-	1	43/3
C17	LGVGIGVGVY	-	-	-	-	-	1/0	-	-	-	-	-	-	-	-	-	50/13	2	51/13
C18	LGVGIRVGVY	-	-	-	-	-	-	-	-	-	-	-	-	-	-	-	1/0	1	1/0
C19	LGIGIGVGVY	-	-	-	-	-	-	-	-	-	-	-	-	-	-	-	10/1	1	10/1
C20	LSIGTGVGGY	-	-	-	-	0/1	-	-	-	-	-	-	-	-	-	-	-	1	0/1
C21	LGVGTGVGAY	0/1	-	-	-	-	-	-	-	-	-	-	-	-	-	-	-	1	0/1
C22	LGVGIGVGXY																2/0	1	2/0
Total	Types	8	5	3	2	4	6	1	1	1	3	1	1	5	2	1	6	16/22	-
	Number	46/12	54/35	34/4	56/14	56/41	80/51	1/0	1/0	3/0	22/8	1/1	1/0	59/19	8/2	1/0	66/16	-	489/203

The number above and below the "/" means the located and unlocated genes.
