# Supplementary material for: Characterization and Classification of LMW-GS Genes at the Glu-3 Locus of Bread Wheat
Source: Int J Mol Sci. 2025 Oct 28;26(21):10482. doi: 10.3390/ijms262110482 (PMC12610552; doi:10.3390/ijms262110482)
Supplement: Supplementary file 1 [file ijms-26-10482-s001.zip › Figure S1.pdf]

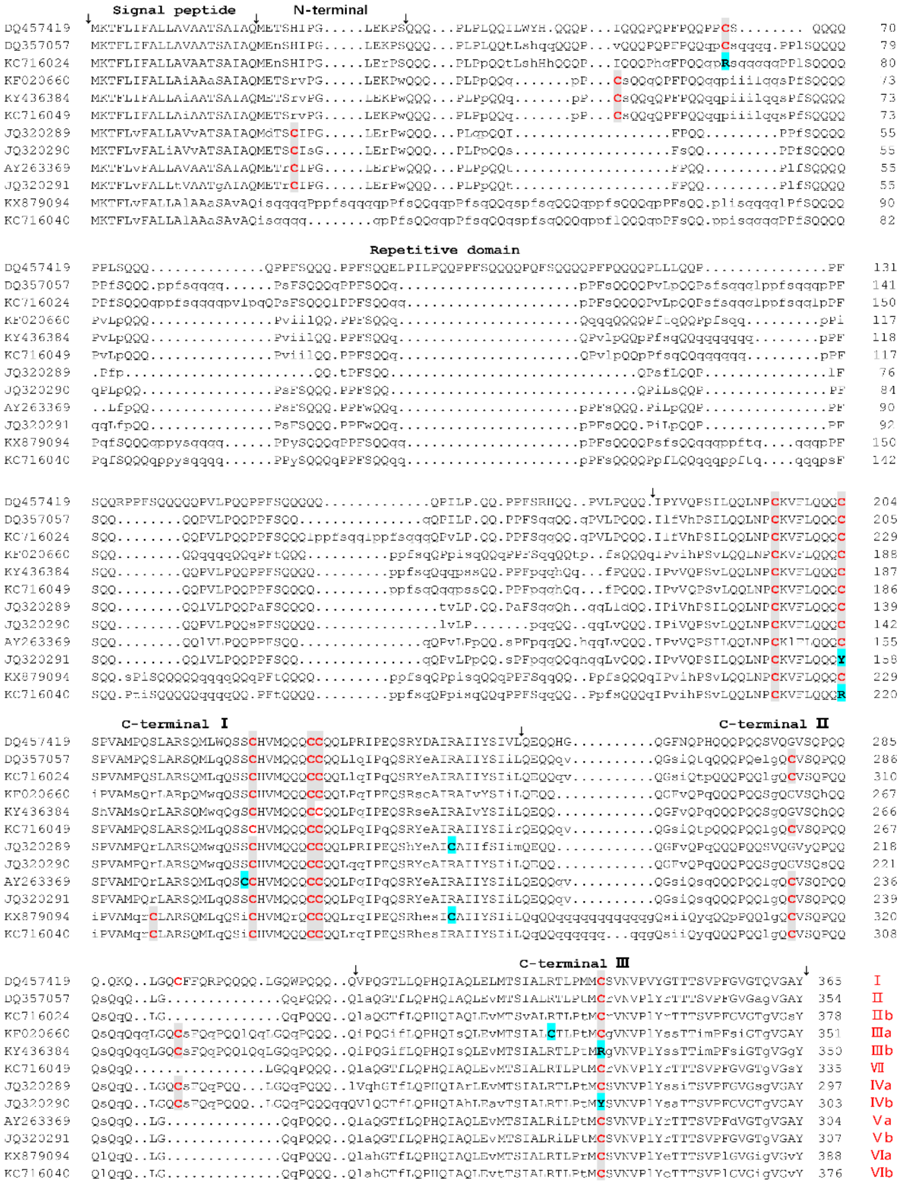

Figure S1. Comparison of classification of the deduced amino-acid sequences among different Glu-3 gene types. The positions of the cysteine residues and their variants are bolded, coloured and shadowed.
