# Supplementary material for: Characterization and Classification of LMW-GS Genes at the Glu-3 Locus of Bread Wheat
Source: Int J Mol Sci. 2025 Oct 28;26(21):10482. doi: 10.3390/ijms262110482 (PMC12610552; doi:10.3390/ijms262110482)
Supplement: Supplementary file 1 [file ijms-26-10482-s001.zip › Table S1.pdf]

Table S1. Classification of LMW-GS genes based on the AA variations of N-terminal domain.

S/N	Types	Accessions of 646 Glu-3 genes	Located	No.	T
N1	MET-SCIP	JQ796685;	GluD3-2	1	
		DQ457420; EU189091; JX877800; JX877816; JX877834; JX877851; JX877868; JX877884; JX877900; JX877920; JX877933; JX877950; JX877964; JX877979; JX877996; JX878012; JX878027; JX878042; JX878058; JX878079; JX878107; JX878119; JX878139; JX878154; JX878164; JX878176; JX878195; KR612300; MH347501; AB062873; JF339155; JF339172; JF339203; KR612306; KR612307; KR612302; KR612305; EU189090; FJ755311; JX878094; KR612301; KR612303; KR612304; MG545988.	GluD3-6	44	46
		EU189092.	GluD3-7	1	
		AB062874; AY695380; AY994361-62; DQ415644; DQ681080; JX828364; JX828375; KC222086; KC222112; MN744854; MN744876.	Unknown	12	12
N2	MET-SCIS	JQ320292.	GluA3-2;	1	
		DQ457416; JX878122; DQ457417; EU189093; KR612296; JF339158; JX877781; JX877802; JX877819; JX877836; JX877870; JX877886; JX877903; JX877923; JX877935; JX877953; JX877966; JX877982; JX877999; JX878015; JX878030; JX878045; JX878061; JX878081; JX878110; JX878142; JX878157; JX878167; JX878179; JX878198; MH347503; JF339174; MN744843; MN744846; MN744858; MN744861; MN744866; MN744869; MN744875; MN744877; MN744888; MN744895; MN744898; MN744904; MN744908; AB062872; MG545989; FJ755314; HM055909; JQ320290; JQ796689; JX878096; KR612297; KY430290.	GluD3-4	54	55
		AY296753; AY994357; AY994359; AY994364; DQ681081; EU329427; GU183486; HQ619900-02; JF339189; JN831407-08; JX828354; KC222085; KC222087; KC222114; KC716050; MN744841-42; MN744851; MN744853; MN744857; MN744862-63; MN744867; MN744878; MN744882-83; MN744887; MN744889; MN744899; MN744902-03; MN744907.	Unknown	35	35
N3	MET-SHIPG	EU189096; FJ755310; JF339165; JX877789; JX877810; JX877827; JX877840; JX877861; JX877877; JX877893; JX877911; JX877943; JX877960; JX877975; JX877988; JX878005; JX878021; JX878036; JX878051; JX878068; JX878104; JX878148; JX878205; MG545993; MH347504; JF339181; JF339197; DQ457419; AB062851; EU189097; FJ755317; JX878085; KR612298; KR612299.	GluD3-5	34	34
		DQ822593; DQ822596; FJ447462; HQ619932.	Unknown	4	4
N4	MET-SHIPS	AB062852; EU369719-20; EU369724-30; EU189089; FJ755306; FJ876823-25; FJ972196; HQ619905; JF339179; JF339194; JF339163; JX877786; JX877806; JX877823; JX877845; JX877875; JX877891; JX877908; JX877928; JX877939; JX877859; JX877957; JX877971; JX878003; JX878019; JX878034; JX878050; JX878066; JX878091; JX878101; JX878115; JX878134; JX878146; JX878127; JX878161; JX878171; JX878183; JX878191; JX878202; JX878212; KR612278-82; MH347498; KY430288.	GluB3-4;	56	56
		HQ619891-98; HQ619904; HQ619906-09; HQ619918.	Unknown	14	14
N5	MET-SRVP	DQ630440; MG574328.	GluA3-7;	2	
		DQ630441; DQ630442; KF020661; KF020662; KJ152528; KJ152530; KJ152531.	GluB3-8;	7	56

		KF020660.	GluB3-9;	1	
		DQ357052; FJ755313; JF339162; JX877785; JX877839; JX877858; JX877874; JX877927; JX877940; JX877958; JX877970; JX877986; JX878002; JX878018; JX878033; JX878065; JX878100; JX878172; JX878184; JX878203; KR612284; MG545991; MH347502; JX877824; DQ357053; EU189098; JX877890; JX877907; JX878049; JX878114; JX878126; JX878160; MG545992; JX878145; AB062865; AB062866; AB062867; KR612283; KR612286; KR612287; KY430289; KY430292; KY430293; KY436384; KY436385; KY436386.	GluD3-1	46	
		FJ028809-10; HQ619899; HQ619924-31; JF339178; JF339193; JX828359; KC222072; KC222074; KC222077; KC222080; KC222083-84; KC222090 KC222113; KC222118; KC716020; KC716025; KC716027-28; KC716030; KC716047-49; KC716051-54; KC716056-61.	Unknown	41	
N6	MET-RCIP	MG574323; MN744845; MN744855;MN744864; MN744897; MG574327; MG574321; MG574322; MG574324; MG574325; MG574326.	GluA3-6;	11	79
		DQ357054; JX877783; JX877821; JX877838; JX877872; JX877905; JX877955; JX877968; JX877984; JX878000; JX878017; JX878032; JX878047; JX878063; JX878112; JX878124; JX878169; JX878200; KJ152532; KJ152533; KR612292; H347500; KC222073; KC222075; KC222116; MN744871; JX877937; KJ152534; KJ152538; DQ357055; JF339160; JX877804; JX877888; KC222089; KJ152537; DQ357056; AB062875; FJ755315; FJ755322; JX877856; JX877925; JX878098; JX878181; MG545990; KC222110; KC222119; KC222121; AY299485; EU189094; AY263369; FJ615309; FJ615310; FJ615311; JQ320291; JQ796688; JX878083; KF020663; KF020664; KF020665; KJ152535; KJ152536; KR612288; KR612289; KR612290; KR612291; MG545996; KY430291; KY436383.	GluD3-2	68	
		DQ681082; EU329426; EU571726; HQ619919-23; JF339176; JF339191; JN831415; JN831417; JN831430; JN831432-34; JX828369; KC222081; KC222091-93; KC222105; KC222107; KC222120; KC716022-23; KC716026; KC716029; MN744844; MN744847; MN744850; MN744852; MN744856; MN744859-60; MN744865; MN744868; MN744870; MN744872; MN744879-81; MN744886; MN744890-91; MN744896; MN744900-01; MN744905-06; MN744909.	Unknown	51	51
N7	MET-RCVP	FJ172533.	GluD3-2	1	1
N8	MET-SCIH	JQ796686.	GluD3-2	1	1
N9	MET-SQIP	JX877826; JX877942; JX877959.	GluB3-11	3	3
N10	MDT-SCIP	FJ549937; JX877803; JX878097; FJ549938; JX877837; JX877871; JX877887; JX877936; JX877967; JX878218; JX878082; JX878132; JX878189; AB062868; AB062869; AB062870; AB062871; FJ755302.	GluA3-2;	18	22
		FJ549946.	GluA3-5;	1	
		JQ796690.	GluD3-2	1	
		JQ320289; JQ796687.	GluD3-8	2	
		EF190322; MN744848-49; MN744873-74; MN744892-94.	Unknown	8	8
N11	MDT-SYIP	JQ320288.	GluA3-2;	1	1
		MN744840.	Unknown	1	1
N12	MEA-RCIP	MG574341.	Glu-A3	1	1
N13	MEN-SHIP	EU369699-703; MH347497.	GluB3-1;	6	59
		EU369704-05; EU369721-23; JX163861-62; MH347496.	GluB3-2;	8	
		AB119006; EU369715-18; FJ755309; KR612277.	GluB3-3;	7	
		AB262661; EU189088; EU369706-10.	GluB3-5;	7	
		EU369711-14; JX877832; JX878089.	GluB3-6;	6	

		DQ357057; JF339167; JX877790; JX877828; JX877841; JX877878; JX877894; JX877912; JX877944; JX877961; JX877976; JX877989; JX878006; JX878037; JX878069; JX878086; JX878206; KR612295; MG545995; MH347499; HQ619911; HQ619917; KR612293; KR612294; MG545994.	GluD3-3	25	
		AB020853; AB020856-58; AB020860-61; HG529977; HQ619903; HQ619910; HQ619912-16; KC716024; KC716055; MN744884; MN744885; MN744910.	Unknown	19	19
N14	IEN-SHIP	DQ357058; FJ755316; JF339182; JF339199; JX877862; JX877929; EU189095; FJ755323.	GluD3-3	8	8
		AB062863-64;	Unknown	2	2
N15	VET-SRVP	KR612285.	GluD3-1	1	1
N16	ISQQQ-	AB062876; AY453154-60; EU189087; EU871816; FJ549928-34; FJ755304; FJ876819-22; JF339169; JF339201; KJ152523; KR612275; KR612276; MH347495. JX877793; JX877830; JX877865; JX877963; JX878075; JX878117; JX878152; JX878174; JX878207; JX878214; KC136287; JX877995; JX877880; JX877899; JX877949; KX879094; JX878092; JX878192; JX877910; JX877987; JX878185.	GluA3-1;	49	66
		AB062877; AB062878; DQ630443; FJ549945; FJ755303; KC136285; KC136286; JX877815; JX877850; JX877798; JX877977; JX878105.	GluA3-4;	12	
		JX877796; JX877797.	GluA3-10	2	
		JX878001; JX878048; JX878064.	GluA3-11	3	
		DQ681079; FJ447464; FJ907548; HQ619933; JF339184; KC222070; KC222088; KC222106; KC716014-15; KC716017-18; KC716040; KC716043-44; KC660353.	Unknown	16	16
	Total	-	-	692	
