# Supplementary material for: Characterization and Classification of LMW-GS Genes at the Glu-3 Locus of Bread Wheat
Source: Int J Mol Sci. 2025 Oct 28;26(21):10482. doi: 10.3390/ijms262110482 (PMC12610552; doi:10.3390/ijms262110482)
Supplement: Supplementary file 1 [file ijms-26-10482-s001.zip › Table S2.pdf]

Table S2. Classification of LMW-GS genes based on the AA variations of C-terminal domain.

S/N	C-ter.	Accession of GenBank	Locus	N-ter.	Total
C1	FGVGT GVGAY	AB062868-71; FJ549937-38; FJ755302; JX877803; JX878097; JX877837; JX877871; JX877887; JX877936; JX877967; JX878082; JX878132; JX878189; JX878218.	GluA3-2	MDTSCIP	18
		JQ320288.	GluA3-2	MDTSYIP	1
		JQ320292.	GluA3-2	METSCIS	1
		FJ549946.	GluA3-5	MDTSCIP	1
		MG574321-27; MN744845; MN744855; MN744864; MN744897.	GluA3-6	METRCIP	11
		EU369699-703; MH347497.	GluB3-1	MENSHIP	6
		FJ972196.	GluB3-4	METSHIPS	1
		AB262661; EU189088; EU369706-10.	GluB3-5;	MENSHIP	7
		DQ357054; JX877783; JX877821; JX877838; JX877872; JX877905; JX877955; JX877968; JX877984; JX878000; JX878017; JX878032; JX878047; JX878063; JX878112; JX878124; JX878169; JX878200; KJ152532; KJ152533; KR612292; MH347500; KC222073; KC222075; KC222116; MN744871; JX877937; KJ152534; KJ152538; DQ357055; JF339160; JX877804; JX877888; KC222089; KJ152537; AY299485; EU189094; FJ615309; FJ615310; FJ615311; JQ320291; JQ796688; JX878083; KF020663; KF020664; KJ152535; KJ152536; KR612288; KR612289; KR612290; KY430291; KY436383.	GluD3-2	METRCIP	52
		JQ796685.	GluD3-2	METSCIP	1
		JQ796686.	GluD3-2	METSCIH	1
		JQ796690.	GluD3-2	MDTSCIP	1
		DQ457416; JX878122; DQ457417; EU189093; KR612296; JF339158; JX877781; JX877802; JX877819; JX877836; JX877870; JX877886; JX877903; JX877923; JX877935; JX877953; JX877966; JX877982; JX877999; JX878015; JX878030; JX878045; JX878061; JX878081; JX878110; JX878142; JX878157; JX878167; JX878179; JX878198; MH347503; JF339174; MN744843; MN744846; MN744858; MN744861; MN744866; MN744869; MN744875; MN744877; MN744888; MN744895; MN744898; MN744904; MN744908; AB062872; MG545989; FJ755314; JQ320290; JQ796689; JX878096; KR612297.	GluD3-4	METSCIS	53
		EU189092; KY430290.	GluD3-7	METSCIP	1
		MG574341.	GluA3-6	MEARCIP	1
		JX878001; JX878048; JX878064.	GluA3-10	**ISQQQQ**	3
		AY296753; DQ681081; EU329427; GU183486; HQ619900-02; JF339189; JN831408; JX828354; KC716050; MN744842; MN744851; MN744853; MN744857; MN744862; MN744867; MN744878; MN744882-83; MN744887; MN744889; MN744899; MN744902-03; MN744907; KC222085; KC222087; KC222114.	Unknown	METSCIS	29
		AY994362; DQ415644; JX828364.	Unknown	METSCIP	3
		DQ681082; HQ619919-23; JN831415; JN831417; JN831430; JN831434; JX828369; KC222091-93; KC716022-23; MN744844; MN744847; MN744850; MN744852; MN744868; MN744872; MN744880; MN744886; MN744890-91; MN744900-01; MN744909; KC222105; KC222081.	Unknown	METRCIP	31
					75

		EF190322; MN744848-49; MN744873-74; MN744892-93.	Unknown	MDTSCIP	7	
		HG529977; MN744884; MN744910.	Unknown	MENSHIP	3	
		KC222106.	Unknown	**ISQQQQ**	1	
		MN744840.	Unknown	MDTSYIP	1	
C2	FGVGA GVGAY	DQ357057; JF339167; JX877790; JX877828; JX877841; JX877878; JX877894; JX877912; JX877944; JX877961; JX877976; JX877989; JX878006; JX878037; JX878069; JX878086; JX878206; KR612295; MG545995; MH347499; HQ619911; HQ619917; MG545994.	GluD3-3	MENSHIP	23	30
		DQ357058; FJ755316; JF339182; JF339199; JX877862; JX877929; FJ755323.	GluD3-3	IENSHIP	7	
		AB062863-64.	Unknown	IENSHIP	2	16
		MN744856; MN744860; MN744865; MN744870; MN744881; MN744896; MN744905.	Unknown	METRCIP	7	
		HQ619903; HQ619910; HQ619912-13; HQ619915-16; MN744885.	Unknown	MENSHIP	7	
C3	FGVGT GVGGY	AB119006; EU369715-18; FJ755309; KR612277.	GluB3-3	MENSHIP	7	15
		EU369711-14; JX877832; JX878089.	GluB3-6;	MENSHIP	6	
		KR612293; KR612294.	GluD3-3	MENSHIP	2	
		KC716026; KC716029.	Unknown	METRCIP	2	8
		AB020853; AB020856-58; AB020860-61.	Unknown	MENSHIP	6	
C4	FGVGT QVGAY	EU189096; FJ755310; JF339165; JX877789; JX877810; JX877827; JX877840; JX877861; JX877877; JX877893; JX877911; JX877943; JX877960; JX877975; JX877988; JX878005; JX878021; JX878036; JX878051; JX878068; JX878104; JX878148; JX878205; MG545993; MH347504; JF339181; JF339197; DQ457419; AB062851; EU189097; JX878085; KR612299.	GluD3-5	METSHIPG	32	32
		DQ822593; FJ447462; HQ619932.	Unknown	METSHIPG	3	4
		AB062874.	Unknown	METSCIP	1	
C5	FGVGT RVGAY	AB062852; EU369719-20; EU369724-30; EU189089; FJ755306; FJ876823-25; HQ619905; JF339179; JF339194; JF339163; JX877786; JX877806; JX877823; JX877845; JX877875; JX877891; JX877908; JX877928; JX877939; JX877859; JX877957; JX877971; JX878003; JX878019; JX878034; JX878050; JX878066; JX878091; JX878101; JX878115; JX878134; JX878146; JX878127; JX878161; JX878171; JX878183; JX878191; JX878202; JX878212; KR612278-82; MH347498; KY430288.	GluB3-4	METSHIPS	55	60
		FJ755317; KR612298.	GluD3-5	METSHIPG	2	
		JX877826; JX877942; JX877959.	GluB3-11	MET-SQIP	3	
		HQ619891-98; HQ619904; HQ619906-09; HQ619918.	Unknown	METSHIPS	14	15
		MN744863.	Unknown	METSCIS	1	
C6	FDVGT GVGAY	AY263369; DQ357056; AB062875; FJ755315; FJ755322; JX877856; JX877925; JX878098; JX878181; KF020665; MG545990; KC222110; KC222119; KC222121; MG545996.	GluD3-2	METRCIP	15	18
		FJ172533.	GluD3-2	METRCVP	1	
		EU189095.	GluD3-3	IENSHIP	1	
		HM055909.	GluD3-4	METSCIS	1	
		EU329426; EU571726; JF339176; JF339191; JN831432-33; KC222107; KC222120.	Unknown	METRCIP	8	9
		JN831407.	Unknown	METSCIS	1	
C7	FGVDT GVGAY	MN744894.	Unknow	**MDT**SCIP	1	1

C8	FSVGT GVGAY	MN744859; MN744879; MN744906.	Unknown	METRCIP	3	5
		MN744876.	Unknown	METSCIP	1	
		MN744841.	Unknown	METSCIS	1	
C9	FGVSA GVGAY	HQ619914.	Unknown	MENSHIP	1	1
C10	FGVGS GVGAY	EU189090.	GluD3-6	METSCIP	1	3
		JQ320289; JQ796687.	GluD3-8	MDTSCIP	2	
		JX828375.	Unknown	METSCIP	1	1
C11	FGVGT GVSAY	AY994357; AY994359; AY994364.	Unknown	METSCIS	3	3
C12	FAVGT GVSAY	AY994361.	Unknown	METSCIP	1	1
C13	FGVGT GVGSY	EU369704-05; EU369721-23; JX163861-62; MH347496.	GluB3-2	MENSHIP	8	
		KC716024; KC716055.	Unknown	MENSHIP	2	5
		DQ822596.	Unknown	METSHIPG	1	
		KC716049; KC716053.	Unknown	METSRVP	2	
C14	FSIGT GVGAY	DQ630441; DQ630442; KF020661; KF020662; KJ152528; KJ152530; KJ152531.	GluB3-8	METSRVP	7	8
		KF020660.	GluB3-9	METSRVP	1	
		FJ028809-10; JX828359; KC222084; KC222074; KC222080; KC222083; KC222113.	Unknown	METSRVP	8	9
		KC222086.	Unknown	METSCIP	1	
C15	FSIGT GVGGY	DQ630440; MG574328.	GluA3-7	METSRVP	2	49
		DQ357052; FJ755313; JF339162; JX877785; JX877839; JX877858; JX877874; JX877927; JX877940; JX877958; JX877970; JX877986; JX878002; JX878018; JX878033; JX878065; JX878100; JX878172; JX878184; JX878203; KR612284; MG545991; MH347502; JX877824; DQ357053; EU189098; JX877890; JX877907; JX878049; JX878114; JX878126; JX878160; MG545992; JX878145; AB062865; AB062866; AB062867; KR612283; KR612286; KR612287; KY430289; KY430292; KY430293; KY436384; KY436385; KY436386.	GluD3-1	METSRVP	46	
		KR612285.	GluD3-1	VETSRVP	1	
		HQ619899; HQ619924-31; JF339178; JF339193; KC222072; KC222077; KC222090; KC222118; KC716020; KC716025; KC716027-28; KC716030; KC716047-48; KC716051-52; KC716054; KC716056-59; KC716061.	Unknown	METSRVP	30	31
		KC222070.	**Unknown**	**ISQQQ**	1	
C16	LGVG RVGAY	AB062873; DQ457420; EU189091; FJ755311; JF339155; JF339172; JF339203; JX877800; JX877816; JX877834; JX877851; JX877868; JX877884; JX877900; JX877920; JX877933; JX877950; JX877964; JX877979; JX877996; JX878012; JX878027; JX878042; JX878058 ;JX878079; JX878107; JX878119; JX878139; JX878154; JX878164; JX878176; JX878195; KR612300; KR612306; KR612307; KR612302; KR612305; JX878094; KR612301; KR612303; KR612304; MG545988; MH347501.	GluD3-6	METSCIP	43	43
		AY695380; DQ681080; MN744854.	Unknown	METSCIP	3	3
C17	LGVGI GVGVY	AB062876; AY453154-60; EU189087; EU871816; FJ549928-34; FJ755304; FJ876819-22; JF339169; JF339201; KJ152523; KR612275; MH347495; JX877793; JX877830; JX877865; JX877963; JX878075; JX878117; JX878152; JX878174; JX878207; JX878214; KC136287;	GluA3-1	ISQQQ	48	51

		X877995; JX877880; JX877899; JX877949; KX879094; JX878092; JX878192; JX877910; JX877987; JX878185.				
		KR612291.	GluD3-2	METRCIP	1	
		JX877798; JX877977.	GluA3-4	ISQQQ	2	
		DQ681079; FJ447464; FJ907548; HQ619933; JF339184; KC222088; KC716014-15; KC716017-18; KC716040; KC716043-44.	Unknown	ISQQQ	13	13
C18	LGVGI RVGVY	KR612276.	GluA3-1	ISQQQ	1	1
C19	LGIGI GVGVY	AB062877-78; FJ549945; FJ755303; DQ630443; KC136285-86; JX877815; JX877850; JX878105.	GluA3-4	ISQQQ	10	10
		KC660353.	Unknown	ISQQQ	1	1
C20	LSIGT GVGGY	KC716060.	Unknown	METSRVP	1	1
C21	LGVGT GVGAY	KC222112.	Unknown	METSCIP	1	1
C22	LGVGI GVGXY	JX877796; JX877797.	GluA3-11	ISQQQ	2	2
Total						692
